# Deciphering the comprehensive microbiome of glacier-fed Ganges and functional aspects: implications for one health

**DOI:** 10.1128/spectrum.01720-24

**Published:** 2025-07-07

**Authors:** Rachel Samson, Shubham Kumar, Syed Dastager, Krishna Khairnar, Mahesh Dharne

**Affiliations:** 1 National Collection of Industrial Microorganisms (NCIM), Biochemical Sciences Division, CSIR - National Chemical Laboratory 29616, Pune, Maharashtra, India; 2 Academy of Scientific and Innovative Research (AcSIR) 550336, Ghaziabad, Uttar Pradesh, India; 3 Environmental Epidemiology and Pandemic Management, CSIR-National Environmental Engineering Research Institute (NEERI), Nehru Marg, Nagpur, Maharashtra, India; Luonnonvarakeskus, Oulu, Finland

**Keywords:** glacier-fed-Ganges, microbiome, special properties, secondary metabolites, bacteriophages

## Abstract

Glacier-fed rivers are significant ecological components of the river catchments, yet their microbial diversity and the associated antimicrobial potential remain underexplored. The Ganges is a glacier-fed river of immense cultural, religious, and ecological significance that supports over 400 million people downstream, providing essential water for agriculture, industry, and daily use. Despite its importance, the microbial community composition and antimicrobial potential, across its relatively pristine origin, remain largely underexplored. One possible explanation for this could be the lower microbial load in the upstream glacier-fed region, which likely results in a reduced DNA yield, insufficient for whole-metagenome sequencing, in contrast to the more biologically diverse and nutrient-rich lower reaches. In this study, we developed an efficient DNA extraction and amplification method using low-input DNA to sequence the microbiome from sediments of the glacier-fed Ganges River in pre-monsoon and post-monsoon over 2 years. Taxonomic and functional diversity of bacterial and viral (phage) communities were analyzed, together with the seasonal variations in their composition. Significant differences in microbial communities were observed in response to seasonal shifts (*P* < 0.05). During the dry season, *Proteobacteria* and *Actinobacteria* were predominant, while *Bacteroidetes* and *Firmicutes* were abundant post-monsoon (*P* < 0.05). The microbiome harbors potential for the biosynthesis of streptomycin, phenylpropanoid, penicillin, and cephalosporins. Bacteriophages from *Podoviridae*, *Myoviridae*, and *Siphoviridae* showed lytic potential against putrefying and pathogenic bacteria. This first comprehensive study on the glacier-fed Ganges River highlights significant seasonal shifts in microbial diversity. The initial insights into the functional profile of the bacterial and phage diversity offer opportunities to explore various natural compounds and enzymes to tackle antimicrobial resistance under the one-health canopy.

**IMPORTANCE:**

This study addresses a knowledge gap by exploring the microbial diversity and antimicrobial potential of the glacier-fed Ganges River across different seasons. The findings reveal various taxa with biosynthetic capabilities for antimicrobial compounds. Additionally, the presence of bacteriophages with lytic potential opens up opportunities for their exploration and application spanning various domains of one health. These findings lay a foundational basis for understanding the unique properties of this riverine ecosystem and offer valuable insights into environmental conservation and the potential to tackle antimicrobial resistance.

## INTRODUCTION

Glacier-fed rivers or streams are a significant component of the earth’s fluvial landscape. Being perennial, they are capable of sustaining the downstream ecosystems, including humans and animals, through the supply of their freshwater all year long (1). Oligotrophy, low water temperatures, and a unique microbiome characterize glacier-fed rivers. Therefore, they are referred to as ecological hotspots and harbor a diverse array of microbial communities, each portraying distinct trophic and biogeochemical functions that largely remain unexplored (2, 3). The diverse microbial communities in glacier-fed rivers drive essential processes such as nutrient cycling, organic matter decomposition, and overall ecosystem functioning. Seasonal shifts in hydrological and nutrient dynamics, particularly the contrast between pre-monsoon and post-monsoon conditions, profoundly influence these microbial assemblages (4). Seasonal variations can affect processes like denitrification and methane production, which have broader implications for water quality and climate regulation (5). Understanding how microbial community structure changes with these seasonal patterns is essential for developing strategies to maintain the ecological balance and resilience of river ecosystems.

Glacial meltwater sediments serve as dynamic microbial habitats, often harboring higher DNA concentrations compared to water columns (6). This significant difference is due to sediments effectively trapping and preserving DNA from various sources, including microorganisms, detritus, and other organic matter, leading to higher DNA concentrations in sediment samples. In contrast, DNA in the water column is more susceptible to degradation and dilution, resulting in lower concentrations (7). Given their depositional nature, these sediments act as long-term reservoirs of microbial diversity, making them valuable sites for studying microbial dynamics in glacier-fed systems.

The Ganges River (Ganga), India’s National River, is one of the most significant glacier-fed rivers of the Himalayan ranges. Originating as the Bhagirathi River at Gomukh, the snout of the Gangotri Glacier, it flows approximately 2,500 km before draining into the Bay of Bengal in the Sundarbans Delta. The upper stretch of the Ganges, spanning approximately 250 km from Gomukh to Rishikesh, remains relatively pristine, with minimal anthropogenic influence compared to the middle and lower reaches. The upper Ganges, particularly in its first 18 km from the glacier snout, remains strongly influenced by glacial meltwater, though it also begins transitioning into a rhithral system with additional hydrological inputs from springs and precipitation further downstream (8). The sediment load in the Ganges upstream is mainly sourced from the glacier-grinding subglacial sediments, monsoon-induced landslides, and debris flow in the Himalayan headwaters (9), as well as climate change-induced glacier retreat and permafrost degradation in the post-monsoon period (10). The Ganges, along its 2,500 km, sustains over 500 million people, serving as a primary source of water for drinking, agriculture, fisheries, and religious practices (11).

The Ganges River has been long known for its non-putrefying and special properties for centuries (12). Ernest Hankin, in 1896, gave an experimental basis demonstrating the antibacterial property of the Ganges River’s water against *Vibrio cholerae* (13). In 1918, Félix d'Hérelle termed the factor conferring this exceptional property to the Ganges as “bacteriophage” (14). Previous studies on the Ganges River isolated fecal coliforms and bacteria resistant to antibiotics and heavy metals (15, 16). Recent research has employed amplicon and shotgun metagenome-based high-throughput sequencing platforms to delineate the resistome at specific locations along the lower stretch of the Ganges River. Zhang et al. (2018) identified intensive allochthonous inputs along the Ganges River, highlighting their substantial impact on microbial community composition and dynamics (17). Reddy and Dubey (18) revealed that the Ganges River water serves as a reservoir of microbes carrying antibiotic and metal ion resistance genes, using a high-throughput metagenomic approach (18). Kumar et al. (19) explored the sediments of the Ganges River, documenting the abundance and diversity of phages, microbial taxa, and antibiotic-resistance genes through a comprehensive metagenomic approach (19). These studies underscore the complex interplay between anthropogenic activities and microbial diversity, with significant implications for environmental and public health.

Additionally, recent studies have expanded our understanding of the physico-chemical parameters and microbial and functional aspects of the Ganges River. Rout et al. (2024) demonstrated the presence of diverse bacterial communities in the Ganges sediments, highlighting not only broad bacterial diversity but also the occurrence of antibiotic-resistant genes, virulence factors, and enzymes potentially involved in the degradation of plastics and xenobiotic compounds (20). Rajput et al. (21) identified functional microbial communities with lignocellulolytic potential along the confluence stretch of the Ganges and Yamuna Rivers, thereby underscoring the capacity of the microbes for biomass degradation (21). In 2020, several investigations advanced our understanding further. For instance, Dwivedi et al. (22) provided evidence for the self-cleansing properties of the Ganges by examining parameters related to mass ritualistic bathing (22). Dutta et al. (23) assessed the impact of the COVID-19 lockdown on the river’s water quality, reporting significant improvements in dissolved oxygen (DO), biological oxygen demand, nitrate, ammoniacal nitrogen, and coliform levels due to a temporary reduction in anthropogenic pollution (23). Behera et al. (24) explored bacterial and fungal diversity in the sediments of the Ganges and Yamuna rivers, emphasizing their potential for bioremediation (24). Dwivedi et al. (22) and Nautiyal (25) provided evidence for the self-cleansing properties of the Ganges, with Dwivedi focusing on mass ritualistic bathing and Nautiyal on the pathogenic *Escherichia coli* O157 (22, 25). Samson et al. (26) investigated the transient influence of the Yamuna River on the microbial communities of the Ganges (26).

Despite these advances, the microbial diversity and functions of the upper/glacier-fed stretch of the Ganges River remain largely unexplored. Under the one health framework, the World Health Organization has classified antimicrobial resistance (AMR) genes and antimicrobial-resistant bacteria as emerging pollutants. Therefore, it becomes imperative to search for newer microbial-based natural products and emerging therapeutic alternatives like bacteriophages and their associated lytic enzymes to reduce the burden of AMR pathogens (27, 28). In our previous study, the microbial diversity, associated resistome, and bacteriophage diversity along the anthropogenically impacted stretch of the Ganges riverine system spanning 1,500 km were studied (29). However, this research primarily focused on the lower stretch of the Ganges and did not address the upper stretch, glacier-fed region. Given its relatively pristine nature and limited anthropogenic influence, studying the upper Ganges provides valuable insights into microbial diversity, phage communities, and their untapped potential to tackle AMR. Additionally, it enables the assessment of seasonal variations in microbial and phage diversity and their ecological implications in a minimally disturbed environment.

To address this knowledge gap, the present study provides the first comprehensive analysis of the representative microbiome in the glacial meltwater sediments of the upper Ganges River. Notably, such sediments are known to serve as long-term reservoirs for microbial communities, accumulating microorganisms over temporal scales due to their depositional nature (4, 5). This study hypothesizes that seasonal shifts in hydrological and nutrient dynamics significantly shape microbial community structure, with distinct differences in microbial and phage diversity between pre- and post-monsoon periods due to variations in water discharge, sediment deposition, and nutrient influx.

**Fig 1:**
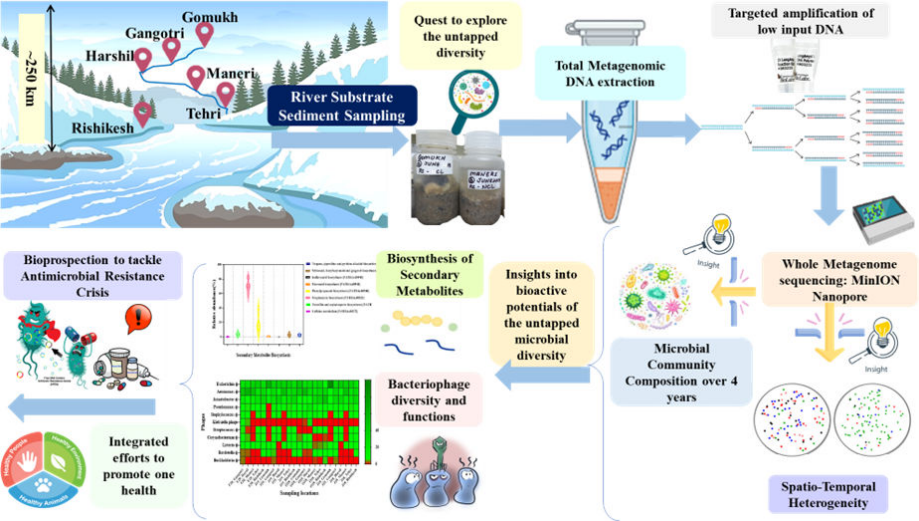
Graphical summary of the metagenomic exploration of microbial diversity in the upper Ganges. River substrate sediment samples were collected from six locations along a ~250 km stretch of the upper Ganges, from Gomukh to Rishikesh. Total metagenomic DNA was extracted to explore untapped microbial diversity, followed by targeted amplification and whole metagenome sequencing using MinION Nanopore. The study investigates microbial community composition over 4 years, uncovering spatiotemporal heterogeneity, bacteriophage diversity, and bioactive potential. Key findings include insights into the biosynthesis of secondary metabolites and the role of bacteriophages in combating antimicrobial resistance, contributing to one health initiative for sustainable bioprospecting.

For the first time, this study introduces a novel approach for the efficient extraction and amplification of metagenomic DNA from glacial meltwater sediments of the upper Ganges. By revealing seasonal microbial shifts, identifying microbes with functional genes related to antibiotic biosynthesis, and highlighting the potential role of lytic bacteriophages in tackling antimicrobial resistance, this research enhances the understanding of microbial dynamics in glacier-fed river systems. These findings contribute valuable insights into the microbiome of a relatively pristine riverine ecosystem and its potential role in addressing antimicrobial resistance, aligning with the one health paradigm (Fig. 1).

## RESULTS AND DISCUSSION

### Microbial diversity measures

Seasonal changes, such as those between pre- and post-monsoon periods, profoundly impact microbial communities by altering temperature, nutrient availability, and hydrological conditions. The pre-monsoon season often has lower water flow and higher temperatures, which can increase microbial activity and diversity. Conversely, the post-monsoon season typically features higher water flow and nutrient dilution, potentially reducing microbial diversity but enhancing the relative abundance of specific taxa adapted to high-flow conditions (30). In this study, we analyzed a representative river sediment microbiome of the Ganges River along its upper stretch, capturing its spatiotemporal variations through three subsamples from six locations along the River substratum. Shannon, Observed, and Simpson’s diversity indices were used to compute the alpha diversity of the representative river sediment microbiome obtained from three subsamples at six locations from the river substratum over 2 years and across two seasons (pre- and post-monsoon). The pre-monsoon season exhibited a greater number of distinct operational taxonomic units (OTUs). However, this observation was not statistically significant. Shannon and Simpson’s diversity indices indicated a relative increase in microbial community richness and evenness in pre-monsoon samples (Fig. S16; supplemental material is found at https://doi.org/10.5281/zenodo.15172343).

The changes in the microbial community structure in response to the seasons (explanatory variable) were comprehended in the Beta diversity using the Bray-Curtis dissimilarity matrix. A higher dissimilarity between the pre- and the post-monsoon samples on axis 1, with 29% variation (Adonis test *P* < 0.05), indicated a significant influence of seasonal variation on the total microbial diversity (Fig. S17; supplemental material is found at https://doi.org/10.5281/zenodo.15172343). Analysis of the viral and phage diversity was done with respect to the season as the explanatory variable and phage diversity as a response variable, using the Shannon function and the Simpson index (Fig. S18 and S19; supplemental material is found at https://doi.org/10.5281/zenodo.15172343). In terms of richness and evenness of the dominant and rare types of bacteriophage, a relatively higher bacteriophage diversity was observed during the pre-monsoon season (Fig. S19; supplemental material is found at https://doi.org/10.5281/zenodo.15172343). Previous studies on species diversity, richness, and evenness have suggested a relatively higher viral diversity in pre-monsoon due to the increased prevalence of available hosts, reduced run-off of river water, and increased nutrient availability (31–33).

### Bacterial community composition

**Fig 2:**
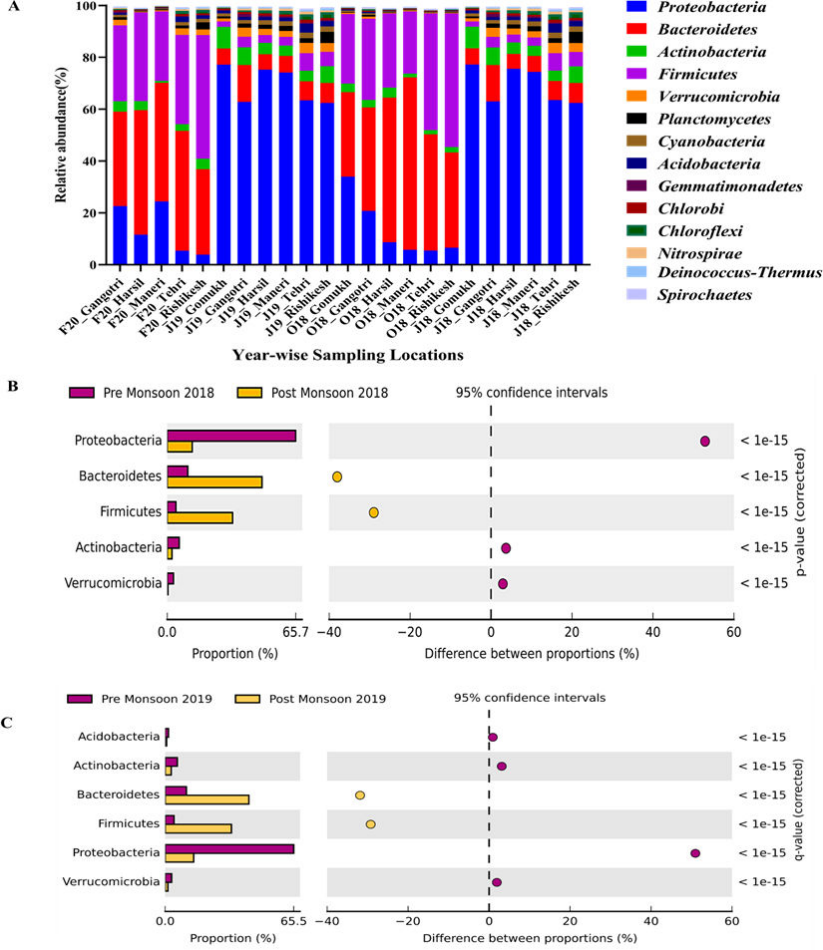
Spatio-temporal variations in the bacterial phyla along the upper stretch of the River Ganges. (**A**) Spatial diversity of top 15 phyla. (**B**) Temporal variations at phylum level in 2018. (**C**) Temporal variations at phylum level in 2019.

Throughout 2 years of sampling encompassing pre- and post-monsoon seasons, a total of 65 bacterial phyla were observed across all the locations. The top 15 most abundant phyla that contribute to the total bacterial diversity are *Proteobacteria*, *Bacteroidetes*, *Firmicutes*, *Actinobacteria*, *Verrucomicrobia*, *Planctomycetes*, *Cyanobacteria*, *Acidobacteria*, *Gemmatimonadetes*, *Chlorobi*, *Chloroflexi*, *Nitrospirae*, *Deinococcus-Thermus*, and *Spirochaetes* (Fig. 2A). This phylum-level overview provides a broad perspective on the microbial community structure in the glacier-fed Ganges and is consistent with findings in other glacier-fed streams (34). During the dry (pre-monsoon) seasons of 2018–2019, *Proteobacteria* (69% ± 6.6%), *Actinobacteria* (6% ± 5.46%), and *Verrucomicrobia* (4% ± 1.23%) were the most prevalent phyla (*P* < 0.05), while in the wet seasons (post-monsoon), *Bacteroidetes* (42% ± 9.16%) and *Firmicutes* (32.8% ± 11.26%) were observed at a higher relative abundance (*P* < 0.05; Fig. 2B and C). These observations are consistent with trends in other aquatic ecosystems, where *Proteobacteria* and *Actinobacteria* are typically more abundant during dry periods (34–37). For example, the copiotrophic nature of *Proteobacteria* is linked to higher nutrient availability and organic carbon during the dry season, a condition further enhanced by increased anthropogenic inputs from trekking and religious activities in the Uttarakhand region (36, 38). *Actinobacteria* phylum is characterized by exceptional stress tolerance, which renders them the ability to survive in extreme habitats such as glaciers, permafrost, and hot springs. The mycelial form of *Actinobacteria* creates favorable conditions in the sediments by mobilization of carbon, nitrogen, and phosphorus pools, thereby contributing to the C, N, and P cycle in the pre-monsoon season (39). Additionally, the prevalence of *Bacteroidetes* and *Firmicutes* in the wet seasons corroborates with the study of Št'ovíček et al., 2017, where an increase in these phyla has been observed during heavy rain and lower temperatures, consequently resulting in a decrease of the relative abundance of *Actinobacteria* and *Verrucomicrobia* (40). The spatial distribution of the top 15 abundant bacteria remains relatively consistent at the phylum level (*P* > 0.99; analysis of variance [ANOVA] with Benjamini-Hochberg [BH] correction).

Glacier-fed riverine systems are dynamic due to temporal fluctuations, especially the temperature that dictates the microbial community structure (34). A distinct taxonomic variation in the bacterial communities was observed in the wake of the seasonal shift. Warmer temperatures can increase microbial metabolic rates, leading to higher decomposition rates and nutrient cycling (41).

**Fig 3:**
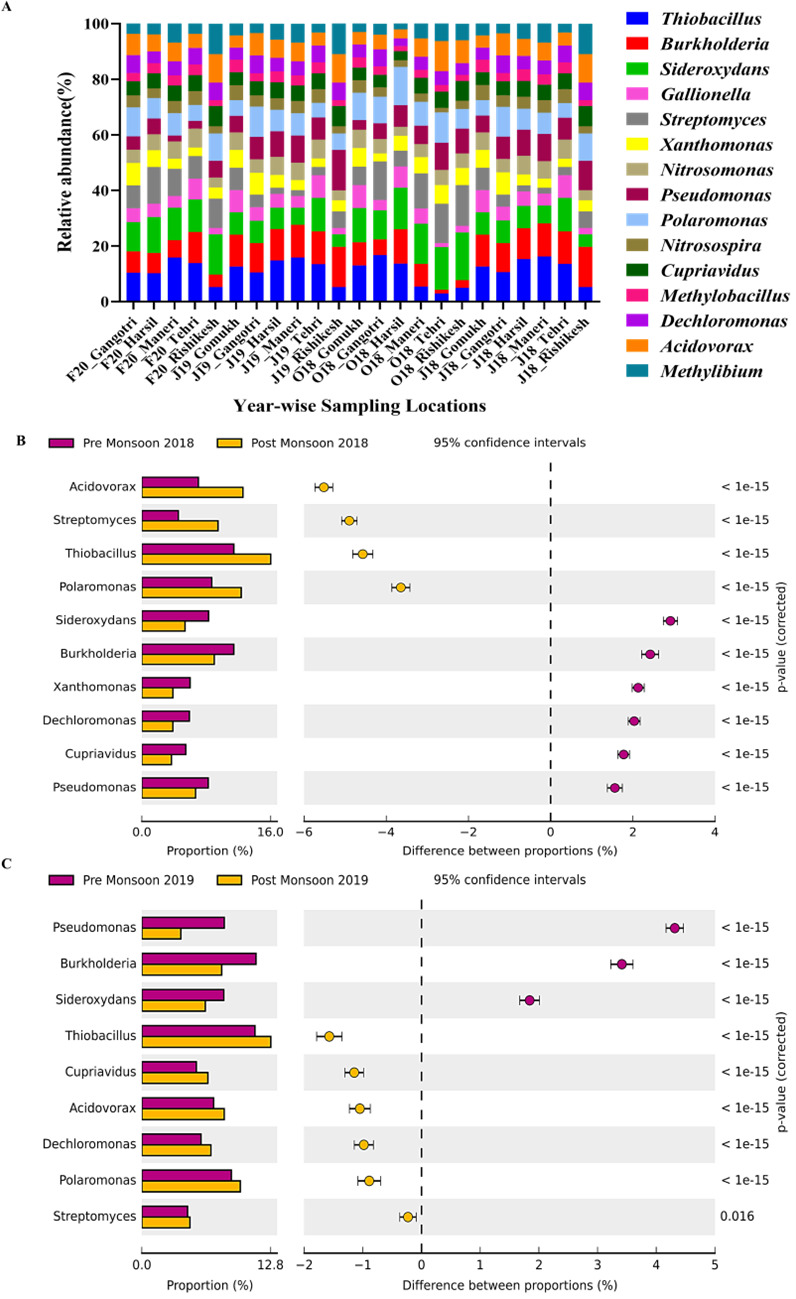
Spatio-temporal variations in the bacterial genera along the upper stretch of the River Ganges. (**A**) Spatial diversity of top 15 genera. (**B**) Temporal variations at genus level in 2018. (**C**) Temporal variations at genus level in 2019.

At the genus level, a predominance of oligotrophic and lithotrophic bacteria, such as *Thiobacillus* (10.2% ± 4.32%), *Sideroxydans* (9.7% ± 3.57%), *Burkholderia* (8.5% ± 4.32%), *Streptomyces* (6.06% ± 4.23%), and *Gallionella* (4.6% ± 2.19%)*,* was observed at all the locations (Fig. 3A). Spatial variation in bacterial diversity at the genus level was not statistically significant (*P* > 0.99; ANOVA with BH correction), indicating a relatively stable composition of key genera across sites (*P* > 0.99). However, seasonal (temporal) variation considerably influenced bacterial diversity (*P* > 0.05; Welch’s *t*-test and Bonferroni correction), with notable shifts in microbial composition between pre- and post-monsoon seasons.

During the pre-monsoon season, *Pseudomonas*, *Burkholderia*, and *Sideroxydans* exhibited higher relative abundances. These genera thrive in environments with lower flow conditions and extended residence times, which provide stable conditions conducive to biofilm formation and nutrient uptake. In contrast, in the post-monsoon season, *Streptomyces*, *Thiobacillus*, *Acidovorax*, and *Polaromonas* showed increased prevalence (Fig. 3B and C). While direct studies linking these genera to high-flow conditions are limited, their metabolic versatility and resilience traits suggest adaptability to dynamic hydrological conditions (42–44).

To further evaluate the effects of spatial and seasonal variability, we performed a linear mixed model (LMM) analysis incorporating OTU as a random effect to account for pseudo-replication. The LMM results confirmed that season and location were not significant predictors of genus-level microbial abundance (*F*-values ≈ 0, *P* > 0.05). This suggests that while observable seasonal shifts occur, they are likely driven by OTU-level variability and unmeasured environmental factors rather than being statistically attributable to season or location alone. Despite the lack of significance in fixed effects, the seasonal trends observed in ANOVA analysis remain relevant, as they align with established ecological principles governing microbial dynamics in glacier-fed riverine systems.

Ecological niches such as glaciers and permafrost are characterized by reducing conditions that support both aerobic and anaerobic microbial communities, including acetoclastic, hydrogenotrophic, methanogenic, sulfate-reducing, Fe(III)-reducing, and denitrifying bacteria (45). The headwaters of the Ganges River, originating from the Gangotri Glacier, experience extreme environmental stressors, including low temperatures and oligotrophic conditions (39). *Thiobacillus* and *Sideroxydans* are known for the anaerobic oxidation of iron and thiosulfate coupled with nitrate reduction in cold temperatures, contributing to the biogeochemical cycling of iron (46). Iron availability plays a crucial role in microbial metabolism, as it is essential for enzymes, cytochromes, and the production of secondary metabolites such as antimicrobials and siderophores (47).

Furthermore, *Burkholderia* and *Streptomyces* were identified as predominant genera in freshwater ecosystems (48, 49). *Streptomyces*, widely known for its complex secondary metabolism, produces a diverse array of bioactive natural products (49, 50). Its presence in stressed environments suggests a competitive survival strategy through antimicrobial compound production (51). Therefore, investigating different *Streptomyces* species in these high-altitude, oligotrophic, and low-temperature environments could facilitate the discovery of novel bioactive compounds. Similarly, the emerging potential of *Burkholderia* in antimicrobial and plant-growth-promoting activities has been highlighted in recent studies (52). Different species of *Burkholderia* are known to have beneficial effects aiding in plant growth, secretion of allelochemicals, and production of antibiotics, as well as siderophores (53).

Additionally, the detection of *Methylobacillus* and *Pseudomonas* is noteworthy. *Pseudomonas* species are well known for their ability to produce heterocyclic antimicrobial compounds (54), while *Methylobacillus*, a methylotrophic genus, has been implicated in phytohormone production that influences plant growth (55). However, *Methylobacillus* remains relatively unexplored in terms of its bioactive metabolite potential. Therefore, the metagenomic sequencing-based insights presented here provide the first comprehensive assessment of microbial community composition and its spatiotemporal variations along the pristine upper stretch of the Ganges River. Despite the non-significance of seasonality and location in our LMM analysis, the observed trends at both phylum and genus levels remain ecologically relevant. These findings offer valuable insights for future microbial bioprospecting and ecological assessments in high-altitude glacier-fed riverine systems.

### *In silico* functional analysis of the metabolic potentials of the microbial community

**Fig 4:**
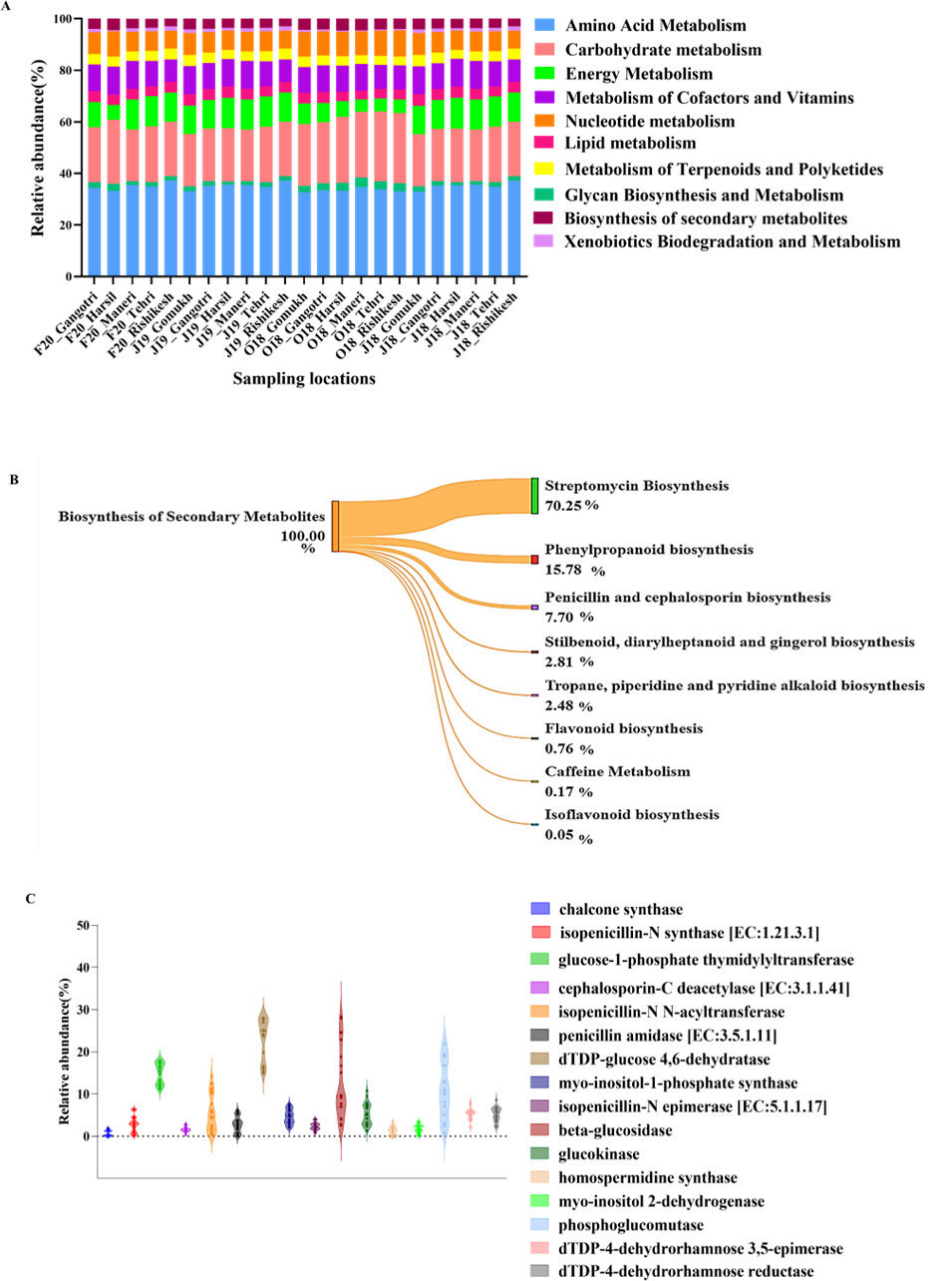
Kyoto encyclopedia of genes and genomes (KEGG)-based classification of reads assigned to the metabolism fraction. (**A**) The sub-metabolic profile with the relative abundance of reads allocated to different types of metabolism. (**B**) Sankey plot illustrating biosynthesis of secondary metabolites. (**C**) Enzymes involved in the biosynthetic gene cluster along the pristine stretch of the Ganges.

The metagenomic analysis of diverse microbial communities in freshwater environments provides a platform for uncovering novel metabolic genes. As glacier-fed rivers are unique ecological niches of microbial diversity, it becomes necessary to investigate the functional potentials of microbes (56). Our analysis revealed several metabolic functions, including the metabolism of amino acids, carbohydrates, energy, glycan synthesis, lipids, cofactors, and vitamins; the biosynthesis of secondary metabolites, terpenoids, and polyketides; and the biodegradation and metabolism of nucleotides and xenobiotics (Fig. 4A). However, we focused our analysis primarily on the genes involved in the biosynthesis of secondary metabolite compounds. The analysis of secondary metabolites identified pathways responsible for producing compounds with antimicrobial properties. A significant fraction was assigned to Streptomycin biosynthesis (70.25% ± 8.11%), followed by phenylpropanoid biosynthesis (15.78 ± 11.93), and biosynthesis of penicillin and cephalosporins (7.79% ± 2.34%; Fig. 4B). The use of antibiotics like streptomycin, penicillin, and cephalosporins has been reported in the treatment of broad-spectrum bacterial infections in wounds, skin, and tissue. Experimental validation supporting the antimicrobial potential of the Ganges River microbiome is scarce (12, 29, 57), and to the best of our knowledge, no studies have yet provided detailed insights into the biosynthetic gene clusters associated with this microbiome. Furthermore, we explored the enzymes and proteins involved in the biosynthesis of secondary metabolites. Analysis revealed a predominance of dTDP-glucose-4,6 hydratase (23.17% ±4.84%), glucose-1-phosphate thymidylyltransferase (14.82 ± 2.47), and betaglucosidase (12.21% ±8.2%) enzymes (Fig. 4C). 4,6-Dehydratases catalyze the initial step in the deoxy sugar biosynthesis pathway. Deoxy (amino) sugars are crucial, especially in the biosynthesis of diverse macrolide antibiotics (58). Another prevalent enzyme, glucose-1-phosphate thymidylyltransferase, is involved in the biosynthesis of dTDP-L-rhamnose, a cell wall constituent of several bacteria, involved in adaptability to harsh environments and virulence. However, L-rhamnose is among the rare monosaccharides that are not commonly encountered in nature. Their limited occurrence makes them valuable, and therefore, the exopolysaccharides of bacteria containing such rare sugars signify an interesting source of isolation (59). Recently, rhamnose-rich bacterial polysaccharides have been reported for their potential biological activities for applications in cosmetics, the food industry, and pharmaceuticals (60). Furthermore, another important enzyme observed in the biosynthetic gene cluster was betaglucosidase, which is fundamental for microbes to adapt to cold environments and plays a crucial role in the carbon flux (61). Additionally, enzymes involved in the synthesis of penicillin and cephalosporins were also observed. Therefore, the presence of enzymes with bioactive potential along the pristine stretch of the Ganges River presents an opportunity for their bioprospecting.

### Archaeal community composition

**Fig 5:**
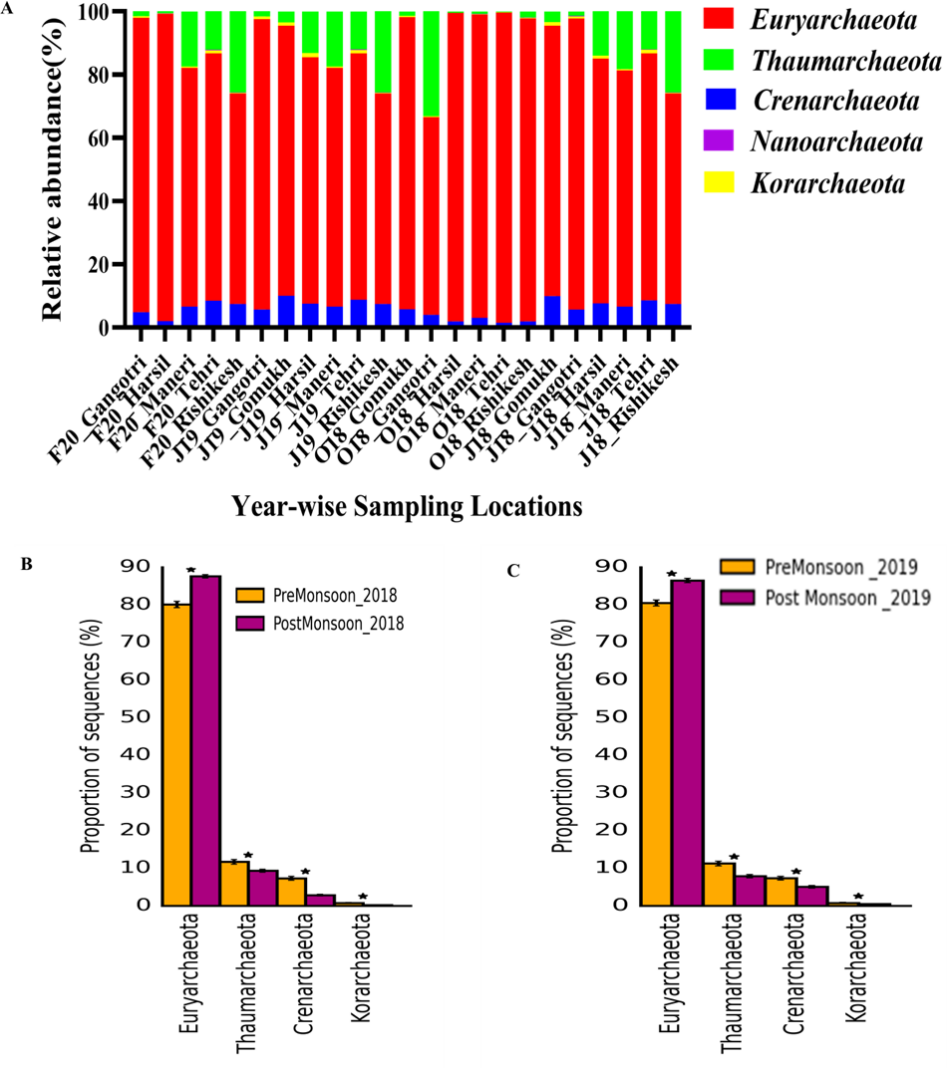
Spatio-temporal variations in the archaeal community composition at the phylum level along the upper stretch of the River Ganges. (**A**) Spatial diversity of top 15 phyla. (**B**) Temporal variations at phylum level in 2018. (**C**) Temporal variations at phylum level in 2019.

The study of archaeal diversity along the upper stretch of the Ganges River reveals crucial insights into its ecological status. The phylum *Euryarchaeota* was observed to be more prevalent in all the samples with a mean relative percentage of (82% ± 11.43%), followed by *Crenarchaeota* (5.35% ± 2.5%) and *Thaumarchaeota* (4.8% ± 10.2%; Fig. 5A). Although the proportion of *Euryarchaeota* was higher in the post-monsoon season, it was the predominant phyla in both seasons (*P* < 0.05; Fig. 5B and C). *Euryarchaeota* comprises methanogenic archaea, anaerobic methanotrophs, and halophilic archaea. The soils of glacier forelands have been regarded as sinks for methane, where the glacier melting affects the methane flux (62). This indicates that glacial meltwater changes the methane sink to a methane source, thereby governing the shift in archaeal diversity from methanogens to methanotrophs (63). This observation highlights the Ganges River’s health in terms of maintaining ecological balance and influencing greenhouse gas emissions.

The *Thaumarchaeota* and *Crenarchaeota* group consists of ammonia-oxidizing archaea, which are essential for nitrification processes in aquatic biomes (64). *Crenarchaeota* phyla comprise hyperthermophilic, sulfur-metabolizing archaea (65). However, members of these phyla have also been reported to be prevalent in different cold habitats, which include high Arctic lakes, Alpine and Antarctic soils, Himalayan cold deserts, and Alpine permafrost (45). *Thaumarchaeota* and *Crenarchaeota* play a crucial role in nitrification, an essential process for water quality. Seasonal variations in these communities can affect the levels of nitrates and other nitrogen compounds, influencing the water quality and availability. Monitoring these microbial shifts helps predict and manage water quality and ensures safe water resources (66, 67).

**Fig 6:**
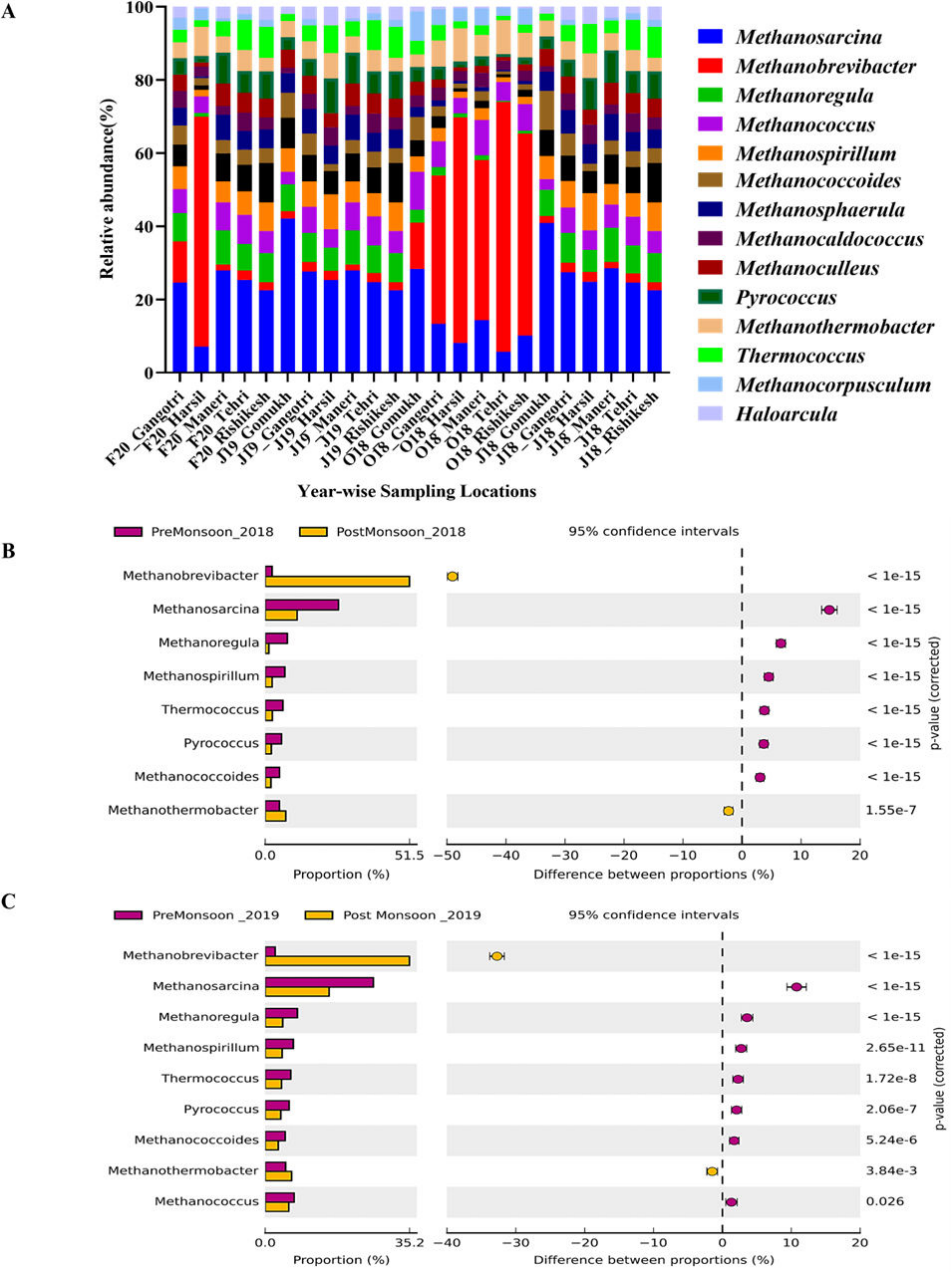
Spatio-temporal variations in the archaeal community composition at genus level along the upper stretch of the River Ganges. (**A**) Spatial diversity of top 15 genera. (**B**) Temporal variations at genus level in 2018. (**C**) Temporal variations at genus level in 2019.

A substantial proportion of the members of *Euryarchaeota* like *Methanosarcina* (20.46% ± 9.45%), *Methanococcus* (6.2% ± 1.82%), and *Methanobrevibacter* (5.8% ± 24.09%; Fig. 6A) was observed. These methanogenic archaea are strictly anaerobic chemolithotrophs, and their substrate utilization is limited to acetate, formate, methylamines, and methanol (65). Additionally, species of these genera have been reported from mesophilic, thermophilic, and hyperthermophilic environments. However, recent studies have shown remarkable stress tolerance (pH, temperature, salinity, desiccation, and oxygen exposure) in methanogenic archaea from cold habitats as opposed to their non-permafrost counterparts (68). Therefore, the information obtained through this study could contribute toward expanding the understanding of the ecology of methanogenic archaea along the Gangotri glacier. Additionally, exploring their adaptive mechanisms can help predict how microbial communities will respond to environmental changes and inform conservation strategies for preserving microbial diversity and ecosystem function. These data could also be helpful for the isolation of potential methanogens as energy carriers for the possible development of alternative energy sources, which, in turn, promotes environmental sustainability beneficial under the one-health canopy.

Interestingly, the seasonal increase in thermophilic and methylotrophic archaeal genera during the dry season highlights the dynamic nature of microbial communities in response to temperature. The proportion of methylotrophic, sulfur-metabolizing, and thermophilic archaeal genera, such as *Methanococcoides*, *Pyrococcus*, and *Thermococcus*, increased in the dry season (*P* < 0.05), indicating the need for increased temperature for their prevalence (Fig. 6B and C). The presence of such thermophiles in such psychrobiotic environments enables opportunities for microbial ecologists to conduct a comprehensive assessment of the novel physiological, adaptive, and molecular mechanisms of these thermophilic archaea for their metabolic potentials to delineate the thermal limits of microbial life (41). To the best of our understanding, this is the first comprehensive report of archaeal community composition and their ecological significance along a ~250 km transect of the pristine stretch of the River Ganges over 2 years. It underscores the importance of microbial diversity in maintaining ecosystem health and stability. Protecting these microbial communities is essential for preserving the natural processes they support and maintaining the overall health of the river ecosystem.

### Viral and bacteriophage diversity and predictive *in silico* functional analysis

Natural environments have a heterogeneous composition of cellular microorganisms, such as protozoa, algae, fungi, bacteria, and archaea, which foster favorable conditions for the sustenance of extensive viral diversity. The comprehensive study of double stranded Deoxyribonucleic acid (dsDNA) viral diversity in the River Ganges reveals the intricate relationships between bacteriophages, microbial communities, and environmental conditions. A notable proportion was assigned to the *Caudovirales* (71.27% ±12.08%) order, followed by unclassified viruses (16.90% ± 10.06%; Fig. S2; supplemental material is found at https://doi.org/10.5281/zenodo.15172343). There are limited studies with riverine ecosystems wherein *Caudovirales* accounted for numerical superiority (69–71). A predominance of *Caudovirales*, which encompasses families of tailed bacteriophages, is indicative of a rich bacteriophage diversity in the Ganges River.

Conversely, in several other studies with freshwater systems, a significant fraction of viral reads consists of unknown or unclassified sequences. This could be attributed to the intrinsic diversity of the virosphere, nucleic acid extraction approach, amplification method, sequencing platform, and the limitations in the availability of the reference databases. This leads to a situation where a considerable fraction of the viral diversity in the environmental metagenomes remains unclassified (72, 73). In our data, yet another considerable fraction was assigned to unclassified viruses. This observation aligns with the detection rates of 6.75% and 28.67% of dsDNA viral abundance in various aquatic metagenomes (69). However, certain virome studies with lakes of the Arctic and Antarctic have shown single stranded Deoxyribonucleic acid viruses and unclassified viruses to be predominant (74). Therefore, to have a holistic understanding of the virosphere (DNA and RNA viruses) of the Ganges River along its pristine stretch, further studies are warranted. Nevertheless, the presence of numerous unclassified viruses in the Ganges River opens avenues for investigating its untapped viral diversity for various potential applications.

**Fig 7:**
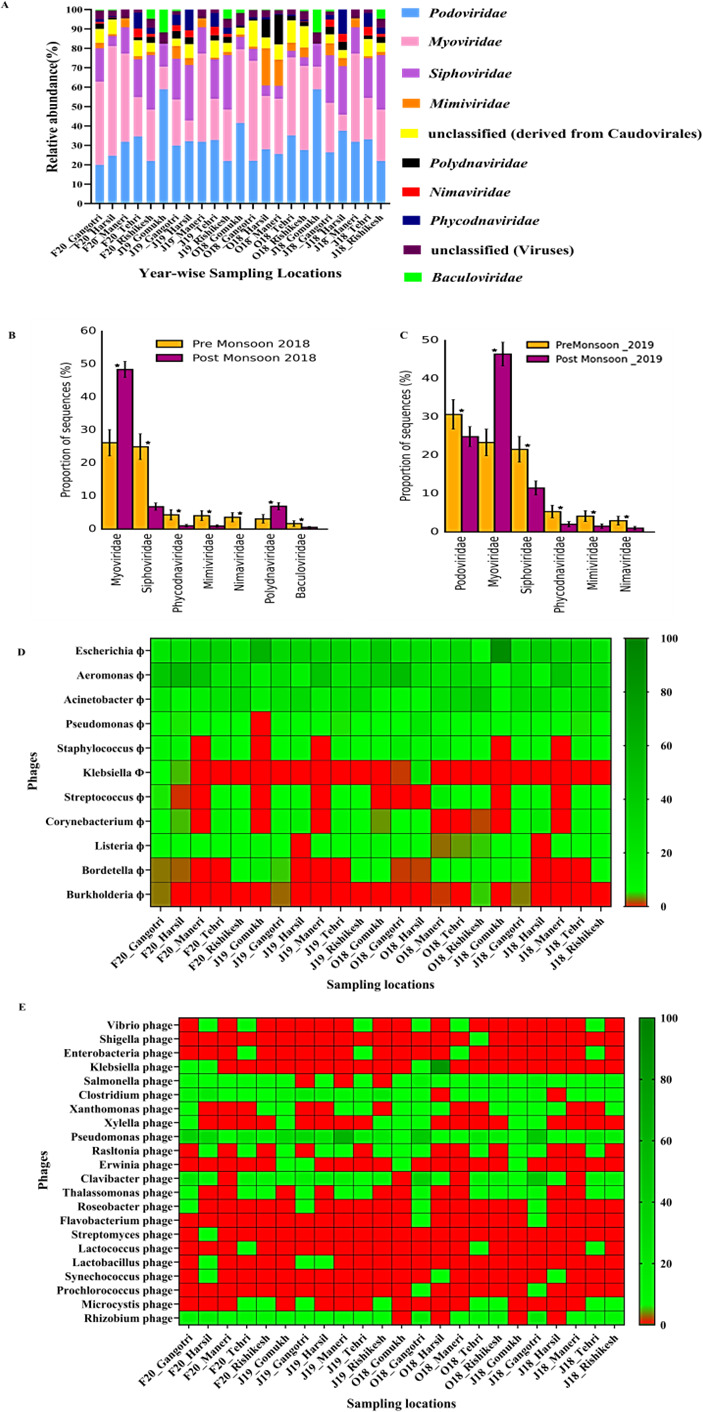
Spatio-temporal variations in the viral community composition along the upper stretch of the River Ganges. (**A**) Spatial diversity of top 10 viral families. (**B**) Temporal variations at the family level in 2018. (**C**) Temporal variations at the family level in 2019. (**D**) Bacteriophage diversity against potentially pathogenic bacteria at the human-animal-environmental interface. (**E**) Bacteriophage diversity against putative putrefying and pathogenic bacteria.

Further classifying the viral reads at the family level revealed a predominance of bacteriophage families like *Podoviridae* (31.70% ±9.99%), *Myoviridae* (30.42% ± 13.61%), and *Siphoviridae* (15.79% ±8.55%), followed by the family of giant viruses infecting protists*—Mimiviridae* (3.89% ±4.35%). The viral community structure was dismally affected along the spatial (location-wise) scale (ANOVA *P* > 0.05 with BH correction; Fig. 7A). However, temporal variations had a substantial influence on the viral diversity (Welch’s *t*-test, *P* < 0.05; Fig. 7B and C). Bacteriophages are known to shape the microbial community structure in the environment through virus-host dynamics. The predominance of bacteriophage families indicated that bacteria were the most important host species. Analysis of the seasonal comparison of viral families indicated an increase in *Podoviridae* and *Siphoviridae* in the pre-monsoon (dry) season. This indicates that the phages belonging to these families primarily comprise members that infect the *Bacteriodetes* and *Firmicutes* groups, as their prevalence is observed to decrease in the dry season. In contrast, the proportion of *Myoviridae* significantly increased during the wet season (*P* < 0.05), suggesting the presence of phages that essentially govern the abundance of the members from the *Proteobacteria* group. This observation could be one of the plausible reasons leading to the decreased prevalence of *Proteobacteria* in the post-monsoon season (Fig.7B and C). Therefore, it could be stated that the phage activity along the pristine stretch of the Ganges River is sequentially triggered by environmental changes, which consequently affects the pool of phages that actively replicate and lyse their cognate hosts across seasons and contribute toward shaping the host community dynamics, ecology, and biogeochemistry of this riverine system.

As the Ganges River is well known for its non-putrefying properties, we analyzed our metagenomic data sets for the presence of bacteriophages from all 23 locations over 2 years. Analysis revealed the presence of bacteriophages against several putrefying bacteria and potentially pathogenic bacteria, affecting human, animal, and environmental health, as illustrated in (Fig. 7D and E). It is noteworthy that the pristine stretch has bacteriophage diversity with untapped potential against the ESKAPEE group of pathogens (*Enterococcus faecium*, *Staphylococcus aureus*, *Klebsiella pneumoniae*, *Acinetobacter baumanii*, *Pseudomonas aeruginosa*, *Enterobacter* sp., and *Escherichia coli*). This could be correlated with the predominance of narrow and broad-spectrum lytic viruses belonging to the *Podoviridae* and *Myoviridae* families at the pristine stretch. However, as each location portrayed substantial phage diversity as classified from the KRAKEN-2 database, we have comprehended their prevalence with illustrative Krona plots for all 23 locations (Supporting file viral and bacteriophage diversity). The presence of a plethora of bacteriophage repertoire for putrefying and potentially pathogenic bacteria along the pristine stretch of the Ganges River could be correlated with its special properties (non-putrefaction). Drawing from this data, the Ganges River emerges as a promising ecological niche to be explored for the isolation of potentially novel bacteriophages along its pristine transect, which could further be used for diverse applications spanning one health.

**Fig 8:**
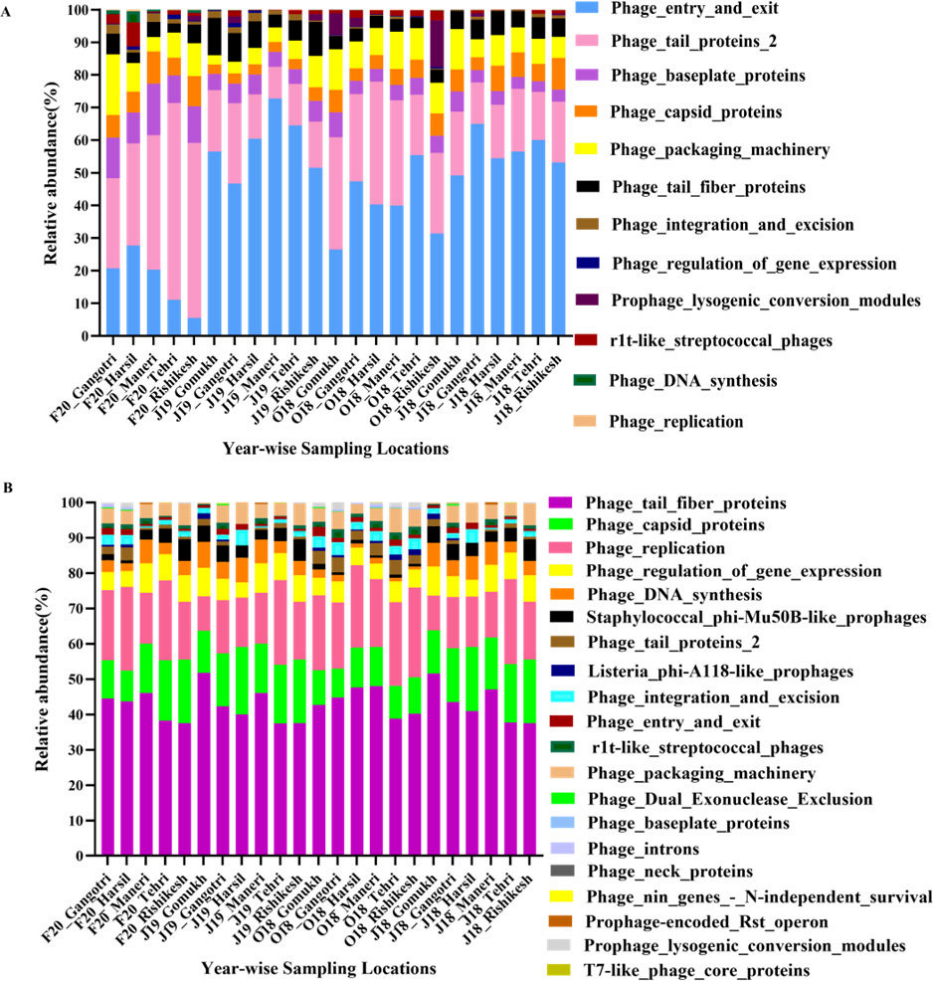
Functional annotation for bacteriophage diversity. (**A**) *In silico* prediction of functional annotations for hits targeting host-phage-related proteins. (**B**) Prediction of phage-associated lytic cassette functions along the upper stretch of River Ganges using MG-RAST.

Additionally, to comprehend phage-related and host-phage-linked functions, predictive *in silico* functional annotation of the data sets was conducted using Subsystems and kyoto encyclopedia of genes and genomes databases in MG-RAST. Analysis revealed the presence of phage-associated functions pertaining to their life cycle, biological process, molecular function, and structural assembly (Fig. 8A). The prevalence of hits against phage-related proteins indicates a robust presence of a host-phage relationship. In our previous study with the anthropogenically impacted (lower) stretch of the Ganges river, which spans ~1,500 km, a predominance of proteins involved in the lysogenic life cycle of phages was observed (29). However, the functional profile in the pristine stretch reflects phage structural and entry and exit-related proteins, indicative of the presence of lytic phages or temperate phages that have initiated the lytic cycle. Previous studies have documented that in ecological niches such as polar regions, cold desert soils, and oligotrophic waters, the virus-to-microbe ratios are higher, thereby favoring the lytic cycle over lysogeny (75, 76). Also, an increase in the availability of resources results in an upsurge in bacterial production in polar regions, ultimately causing a switch of temperate viruses from lysogeny to lytic lifestyle (77–79).

In our previous study, where the anthropogenically impacted stretch was comprehended for its representative river sediment microbiome, from three subsamples at 15 locations from the river substratum, a distinct pattern of viral diversity was observed (Welch’s *t*-test, *P* < 0.05; Fig S20A and B; supplemental material is found at https://doi.org/10.5281/zenodo.15172343). The predominant viral families there were giant viruses that infect protists, i.e., *Mimiviridae* (31.68% ± 11.16%) and phages from *Siphoviridae* (primarily temperate phages; 19.09% ±5.06%), *Myoviridae* (broad-spectrum lytic phages; 11.23% ±4.44%), and *Phycodnaviridae* (algae-infecting phages; 7.38% ±2.83%) families. Moreover, a predominance of integrases, excisionases, and prophage sequences was observed, suggesting the presence of phages with a temperate lifestyle (29). Therefore, this riverine system offers a unique niche with a substantial eco-diversity of its autochthonous bacteriophages to gain insights into their fundamental, ecological, and applicative aspects. Interestingly, our investigation also revealed hits for sequences associated with phage-associated lytic activities (including endolysin, endopeptidases, lysozyme, and amidase; Fig. 8B). These enzymes hold potential for their use in therapy, diagnostics, and various biotechnological applications. The study’s findings on the prevalence of lytic cycle-associated proteins in the pristine stretch of the River, compared to lysogenic life cycle proteins in anthropogenically impacted stretches, suggest that environmental conditions influence phage life cycle strategies. This knowledge can help predict how viral communities may respond to environmental changes. Given that this study serves as an initial exploration of phage diversity and its associated functions, further research is essential to fully harness the potential of these phage communities for ecological, biotechnological, and therapeutic applications under one health framework.

Overall, the present study provides the first comprehensive report that underscores the compositional heterogeneity of microbial diversity along a spatiotemporal gradient of the pristine stretch of the Ganges River. The strength of the study lies in the approach used to detect the microbiome and its antimicrobial potential along the glacier-fed pristine stretch containing low-DNA samples. The results highlight the microbial diversity of this glacier-fed river as a repository with functional potential for the synthesis of secondary metabolite compounds known for their antibacterial effect. Therefore, these data could be used as a roadmap for bioprospection and isolation of microbes with antibacterial potentials. Additionally, the bacteriophage diversity with lytic potentials toward putative putrefying and potentially pathogenic bacteria was observed. The significance of this research is underscored by its alignment with the “one health” concept, which emphasizes the interconnectedness of human, animal, and environmental health. The unique microbial diversity and antimicrobial potential of the glacier-fed Ganges River, as revealed in this study, provide insights to be explored further to tackle AMR pathogens. However, further studies that demonstrate the bioactive potentials of microbiota, including phages, are warranted to have a holistic understanding of the promising capacities of untapped microbial diversity and their contribution toward the special properties of the Ganges.

## MATERIALS AND METHODS

### Sampling and study site details

**Fig 9:**
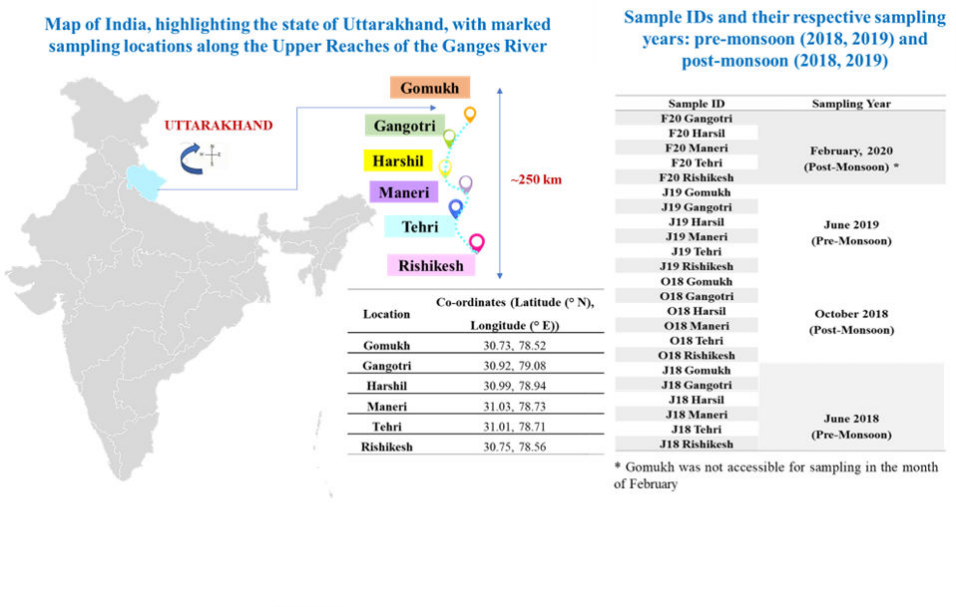
Schematic illustration of the upper reaches of the Ganges River in Uttarakhand, India, showing sampling locations (Gomukh, Gangotri, Harshil, Maneri, Tehri, and Rishikesh). The figure includes sample IDs and their respective sampling years across two seasons: pre-monsoon (2018 and 2019) and post-monsoon (2018 and 2019) over 2 years (map not to scale).

The current investigation focused on a ~250 km long transect of the River Ganges for its microbiome and its antimicrobial potential. A total of 69 sediment samples (depth ~40–50 cm) were collected from six locations over 2 years (2018–2020; Fig. 9). The entire course of the river Ganges in our study was broadly divided into two different stretches. The upper transect was from Gomukh (30.73°N, 78.52°E) to Rishikesh (30.75°N, 78.56°E; ~250 km), while Haridwar (29.94°N, 78.16°E) to Bhagalpur (25.15°N, 87.42°E; ~1,500 km) marks the lower stretch. The lower stretch is characterized by the River Ganges debouching from the Himalayan region into the Gangetic plains and sufficing a diverse civilization alongside its basin.

The upper reaches of the Ganges River, particularly in the state of Uttarakhand, India, are characterized by a sparse population due to the challenging mountainous terrain and harsh climatic conditions. The inhabitants of this region primarily belong to indigenous communities such as the Bhotiya, Jaunsari, and Rajis. The Bhotiya people, for instance, are traditionally semi-nomadic pastoralists residing in the higher altitudes near the Indo-Tibetan border (80). In contrast, the lower stretches of the Ganges River, encompassing the expansive Indo-Gangetic plains in states like Uttar Pradesh, Bihar, and West Bengal, are among the most densely populated areas in the world. This high population density is attributed to the fertile alluvial soils, abundant water resources, and favorable agricultural conditions that have supported extensive human settlement and intensive farming practices over centuries (81). The demographic contrast between the upper and lower reaches of the Ganges provides insight into how geography and environment influence human habitation patterns along the river’s course. The sampling strategy was similar to our previous studies, which involved the collection of sediment samples from the River in triplicates from the left, center, and right banks (26).

In 2019, Uttarakhand experienced significant deviations in rainfall patterns. The post-monsoon season (October 2019 to January 2020) recorded 114.6 mm of rainfall, which is 89% above the normal average (82, 83), as a result of which the post-monsoon sampling for 2019 was carried out in February 2020. *In situ* measurements of the environmental parameters like pH, DO, electrical conductivity, and temperature were carried out using respective probes (Table S1, supplemental material is found at https://doi.org/10.5281/zenodo.15172343). The samples collected were preserved on dry ice and immediately transported to the laboratory for further investigation.

### Metagenomic DNA extraction and shotgun sequencing

Prior to the DNA extraction, the sediment samples from the left, center, and right banks of the River were pooled together according to the locations. Subsequently, the pooled samples from each location were subjected to DNA extraction. Efficient extraction of DNA from sediments of glacier-fed aquatic ecosystems with low microbial load is challenging (37, 84). Therefore, we have evaluated 14 different methods for the extraction of total metagenomic DNA from the sediments of the pristine stretch of the River Ganges (Supporting Information: Text S1 to S14, Fig. S1 to S13, Results and discussion for Evaluation of the optimal DNA extraction method [S1–S14]; supplemental material is found at: https://doi.org/10.5281/zenodo.15172343). Among the various methodologies assessed, the DNeasy PowerMax Soil Kit (Qiagen) approach with certain modifications was considered as a method of choice, evidenced by its desirable DNA ratio (*A*_260_/*A*_280_ of 1.7–1.9) and a substantial DNA concentration of 50–100 ng/µL (Fig. S14; supplemental material is found at https://doi.org/10.5281/zenodo.15172343).

The extracted DNA samples were amplified using three different amplification methods: random amplification by Repli G (Qiagen), MALBAC (Yikon Genomics), and targeted amplification using Long Amp enzyme coupled with 1-D low input genomic DNA with PCR (SQK-LSK109 with EXP-PCA001) and Oxford Nanopore Technologies. To avoid potential bias resulting from the amplification process, we initially evaluated one of the lower stretch samples from our previous study (29), having sufficient DNA concentration for direct sequencing on the MinION platform. Subsequently, we mimicked its concentration to that of the upper stretch samples by diluting it to 100 ng and then amplifying it with the amplification methods mentioned above. This approach allowed us to compare the taxonomic profiles of the original sample vs the amplified ones. Details of this approach have been documented in the Supporting Information (Text S15: Comparison of amplification methods, Results and discussion for the comparison of the amplification methods using a control sample, Fig. S15, Table S2 to S4; supplemental material is found at https://doi.org/10.5281/zenodo.15172343).

Based on the results obtained from the lower stretch sample, we selected Long Amp enzyme coupled with 1-D low input genomic DNA with PCR (SQK-LSK109 with EXP-PCA001) and ONT as the method of choice for amplification of the DNA for the upper stretch samples. Briefly, in this process, 100 ng of DNA from each of the samples was end-prepped using Ultra II End-Prep buffer and Ultra II End-Prep enzyme mix (New England Biolabs [NEB]). The resulting DNA was quantified using a Qubit fluorimeter (Thermo Fisher Scientific). The end-prepped DNA fragments were further taken for adapter ligation using a PCR Expansion kit (EXP-PCA001). The adapter-ligated DNA was then amplified using Long Amp Taq2X master-mix (NEB), and the Ampure XP beads (Beckman Coulter) were used for the purification of the resultant amplified product.

Furthermore, the amplified DNA samples were indexed with a 1D Native barcoding genomic DNA kit (EXP-NBD103 and EXP-NBD 113). The library was prepared using a Ligation Sequencing Kit (SQK-LSK109) with certain modifications, as mentioned in our previous study (29). The final library was loaded onto the FLO-MIN-106D-R9 flow cell (ONT) placed in the MinION sequencing platform, and the run was continued up to 56–72 hours.

### Data processing and quality check

Processing of the raw reads and their quality assessment was carried out as per our previous study (29). Briefly, base-calling of the raw reads was carried out with Albacore (v2.3.4). Subsequently, the fastq files were subjected to qcat software v1.1.0 (ONT) for demultiplexing. The adapter sequences were trimmed with the Porechop tool (v0.2.1). The data sets were normalized with the DESeq2 package. The data quality and stats were assessed using Nanostats in NanoPlot. The normality of the data sets was evaluated with the Shapiro-Wilk normality test (*P*-value ≤ 0.05), confirmed using a Q-Q plot. Furthermore, the data were rarefied using the rarefy function in R. These rarefied data sets were used for downstream taxonomic and functional analysis.

### Microbial community structure and statistics

The metagenomic data sets were annotated for taxonomic and functional composition using default parameters in MG-RAST (*E* value: 1 × *e*−5 and 60% of sequence identity). Additionally, the KRAKEN-2 tool was used for improved taxonomic resolution (10). GraphPad Prism v9.5 was used to prepare illustrations. Differences in the abundance of microbial diversity with respect to seasonal changes were evaluated using Welch’s *t*-test with Bonferroni correction (*P*-value ≤ 0.05) in STAMP (Statistical Analysis of Taxonomic and Functional Profiles) software v2.1.3 (85). ANOVA with BH correction was performed to identify significant differences in the microbial community using GraphPad Prism v10.2.

Taxonomic diversity over a spatio-temporal scale was ascertained using alpha and beta diversity measures. Prior to the analysis, the removal of singletons was ascertained from the OTU file, and the OTU table was rarefied to ensure equal sampling depth. Alpha diversity metrics of observed OTUs, Shannon diversity index, and Simpson’s index were computed with the phyloseq package in R (86). Beta diversity analysis using the Bray-Curtis dissimilarity matrix was carried out to understand compositional differences between the samples. The significance of the dissimilarity measure was determined through analysis of similarities and permutational multivariate analysis of variance (Adonis test *P*-value < 0.05). The similarities and differences between the samples with reference to the season (explanatory variable) and microbial diversity of the sampling locations (response variables) were visualized using the principal coordinate analysis plot.

Additionally, to account for pseudo-replication in our data set and to ensure that the observed microbial patterns are not biased by the non-independence of samples, an LMM analysis was performed to evaluate the effects of spatial and seasonal variations on microbial diversity. The LMM was implemented in R (v4.4.2) using the lme4 and lmerTest packages. The sampling location and season were treated as fixed effects, and OTU-level variations were incorporated as a random effect to account for repeated measurements across different sites and seasons.

### *In silico* predictive functional annotation for bioactive potentials of untapped microbial diversity

The fastq files uploaded in MG-RAST were used for functional annotation. The metabolic potentials of microbes in each data set were analyzed by assigning kyoto encyclopedia of genes and genomes orthology numbers obtained from known reference hits, and annotation of the metabolic pathways with respect to the biosynthesis of secondary metabolites was done using kyoto encyclopedia of genes and genomes pathway analysis. Additionally, predictive *in silico* functional analysis of the data sets to comprehend phage-related functions was achieved using Subsystems and kyoto encyclopedia of genes and genomes databases in MG-RAST.

## Data Availability

The metagenomic data of this study are publicly accessible through MG-RAST under the project titled "Ganga_UpperStretchdata_NCL2022." Additionally, these data have been submitted as bio project PRJNA1101206 to the Sequence Read Archive (SRA) portal. The accession IDs and sequence details for all the data sets from both MG-RAST and SRA have been summarized (Supporting file_Metagenomes details US; supplemental material is found at https://doi.org/10.5281/zenodo.15172343).
